# A computational model of gene expression reveals early transcriptional events at the subtelomeric regions of the malaria parasite, *Plasmodium falciparum*

**DOI:** 10.1186/gb-2008-9-5-r88

**Published:** 2008-05-27

**Authors:** Matthias Scholz, Martin J Fraunholz

**Affiliations:** 1 Competence Center for Functional Genomics, Ernst-Moritz-Arndt University, Friedrich-Ludwig-Jahn Strasse, D-17487 Greifswald, Germany

## Abstract

**Background:**

The malaria parasite, *Plasmodium falciparum*, replicates asexually in a well-defined infection cycle within human erythrocytes (red blood cells). The intra-erythrocytic developmental cycle (IDC) proceeds with a 48 hour periodicity.

**Results:**

Based on available malaria microarray data, which monitored gene expression over one complete IDC in one-hour time intervals, we built a mathematical model of the IDC using a circular variant of non-linear principal component analysis. This model enables us to identify rates of expression change within the data and reveals early transcriptional events at the subtelomeres of the parasite's nuclear chromosomes.

**Conclusion:**

A delay between subtelomeric and central gene activities suggests that key events of the IDC are initiated at the subtelomeric regions of the *P. falciparum *nuclear chromosomes.

## Abstract 2

A mathematical model of the intraerythrocytic developmental cycle identifies a delay between subtelomeric and central chromosomal gene activities in the malaria parasite, *Plasmodium falciparum*.

## Background

The protozoan parasite *Plasmodium falciparum *causes malaria in humans. The life cycle of *Plasmodium *includes multiple stages of development that take place in the mosquito vector and, upon infection of humans, in liver and red blood cells (RBCs). In erythrocytes, the malaria parasites undergo an asexual reproductive cycle (the intra-erythrocytic development cycle (IDC)), which is responsible for pathogenesis in humans. After invasion of RBCs, merozoites establish a ring-like form within the parasitophorous vacuole, which develops to form the trophozoite stage during which the parasite is feeding on hemoglobin. After multiple replications of the parasite genome, trophozoite cell components are packaged into multiple schizonts and, upon rupture of the RBC membrane, mature merozoites are released, each of which will re-initiate a new IDC. Bozdech *et al*. [1] and Llinas *et al*. [2] presented highly time-resolved microarray analyses of the transcriptomes of *P. falciparum *strains HB3, 3D7, and Dd2 during their IDC. In these analyses most genes were shown to behave in a sinusoidal fashion, with one peak of strong up-regulation and one dip in the expression data. This cyclic behavior prompted us to analyze these transcriptome data in order to identify genes that involve a circular component in data space. To model the infection cycle and obtain the rate of change for each gene at any time, we built a mathematical model of the IDC by using a non-linear dimensionality reduction technique based on neural networks, termed circular principal component analysis (PCA) [3]. The model provides continuous and noise-reduced approximations of gene expression curves in a multivariate manner and thus gives the amount of expression and the rate of change (slope) at any time, including interpolated times. We used circular PCA for visualizing gene up- and down-regulation on the 14 chromosomes of the *P. falciparum *nuclear genome. As a result, we observed that subtelomeric regions show a behavior that is clearly distinct from central chromosomal regions: we found that some subtelomeric genes or regions are strongly up-regulated prior to a general/global up-regulation of genes in more central chromosomal regions. This suggests that genes in subtelomeric regions or the subtelomeres themselves may play a role in controlling genes of internal chromosomal regions.

## Results

To model the infection cycle of the *P. falciparum *parasite during its intra-erythrocytic development, we built a mathematical model of the IDC. Genome data for our analysis were obtained from PlasmoDB [4]. Expression data of Bozdech *et al*. [1] and Llinas *et al*. [2] were obtained from the laboratory's web site (see Materials and methods). The full gene expression dataset consisted of 4,859 genes (represented by 7,091 oligonucleotides on the microarray and 46 time points for strain HB3, 53 time points for strain 3D7, and 50 time points for strain Dd2). The datasets were filtered to remove genes whose expression was either constantly 'on' or 'off' or too noisy to be analyzed in the subsequent calculations. Pre-filtering reduces the gene set used in our analysis to 3,639 genes (HB3), 2,419 genes (3D7), and 2,718 genes (Dd2). Additional data file 1 lists genes that have been included in the analysis. To reduce the dimensionality of the dataset, we used a neural network implementing circular PCA, a special case of non-linear PCA (NLPCA) [5].

### The gene expression data of the IDC form a circular structure caused by variation over time

**Figure 1:**
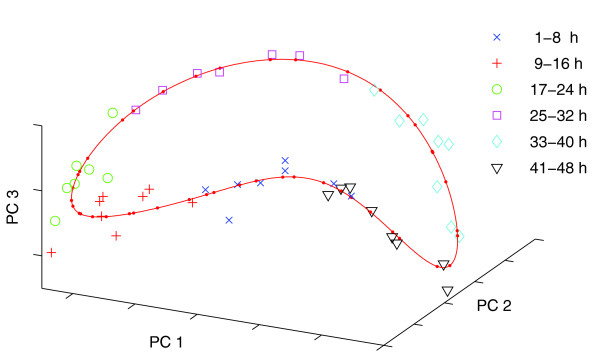
Circular component. The gene expression data of the IDC form a circular structure caused by variation over time. Circular PCA is used to identify a circular component (red line) that approximates the data and, hence, provides a noise-reduced and continuous model of the IDC. The component describes a curve located in the high-dimensional data space given by all genes. The component and the original data are visualized by plotting them into the reduced space given by the first three (linear) principal components (PC1-3) of standard PCA.

**Figure 2:**
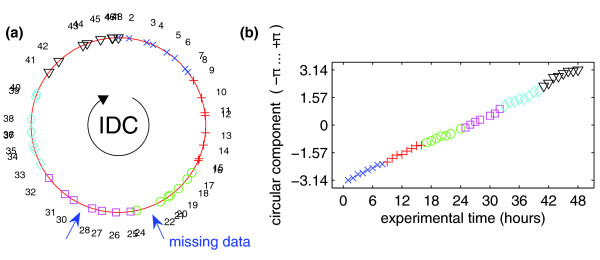
Time component versus original experimental time. **(a) **The identified circular component (red line) with marked positions on the component corresponding to the observed data. The overlap between the first and last observations is a result of component analysis and not explicitly supplied in advance. Since the first time point (1 hour) matches the last observation (48 hours), the data indicate that one cycle takes about 47 hours. The expected gaps for missing observations at 23 and 29 hours are also apparent (arrows). Other gaps might be caused by technical variation. **(b) **Plotting original (experimental) time points against their corresponding component values confirms that the identified component represents the trajectory of the data over time and, therefore, can be regarded as the time component.

By using circular PCA we were able to identify a circular component (Figure 1, red line), which approximates the expression data and, hence, provides a noise-reduced and continuous model of the IDC. The component describes a curve located in the high-dimensional data space given by all genes. Figure 1 visualizes the component and the original data by plotting them into the reduced space given by the first three (linear) principal components (PC) of standard PCA. To identify the contribution to the cyclic component, we plotted the components of the observed data with respect to time points (Figure 2a). An overlap between first (t = 1 h) and last observation (t = 48 h) further suggests that one development cycle of the investigated malaria parasites lasts about 47 hours. This value is a result of component analysis and has not been supplied in advance. The expected gaps for missing observations at 23 and 29 hours are also identified by our algorithm (HB3 dataset [1]). Thus, plotting the original (experimental) time points against their corresponding component values confirms that the identified component represents the trajectory of the data over time and, therefore, can be regarded as the time component (Figure 2b).

### Early transcriptional events of the IDC are predominantly within the subtelomeric regions

**Figure 3:**
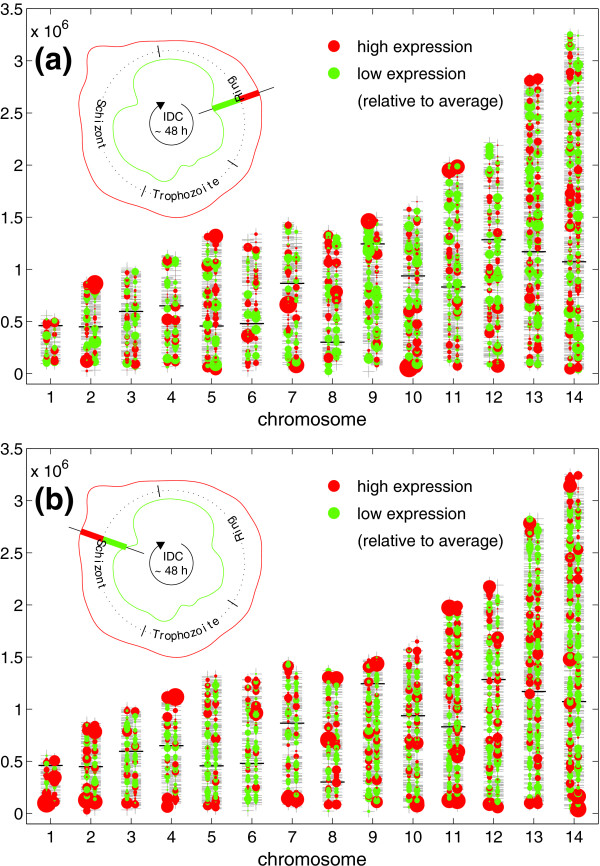
Snapshots of gene expression during the IDC. Expression ratios indicate early transcriptional activities at *P. falciparum *subtelomeres. Shown are two frames of an animation of expression levels detected on the chromosome loci during the IDC: **(a) **10 hours and **(b) **40 hours post-infection. Red dots indicate high and green dots low expression levels as determined by intensity ratios. Note the accumulation of highly expressed genes at the chromosomal ends. Upper left corner of each frame: 'infection timer' where the red and green curve represent the number of genes having their highest (red) or lowest (green) expression at the respective time (density curves over time of maxima/minima of all genes weighted by the gene intensity; see also Materials and methods).

Our model provides continuous and noise-reduced approximations of gene expression curves in a multivariate manner and thus gives the amount of expression and the rate of change (slope) at any time in the time course, including interpolated times. Figure 3 shows two frames (10 h and 40 h post-infection, respectively) of an animation of the expression levels of the 14 nuclear malaria chromosomes during the IDC of *P. falciparum *HB3. The upper left corner of each figure displays an 'infection timer' representing the number of genes having their highest (red) or lowest (green) expression at a certain time point. The genes with strong (red dots) or weak (green dots) expression are indicated, whereas the diameter of the dot indicates the expression level (for example, a large green dot means very low expression ratio). As a result, we observed that subtelomeric regions (termini of the linear chromosomes) show a behavior that is clearly distinct from central chromosomal regions. An animation of the change of expression ratios throughout the IDC is provided in Additional data file 5.

**Figure 4:**
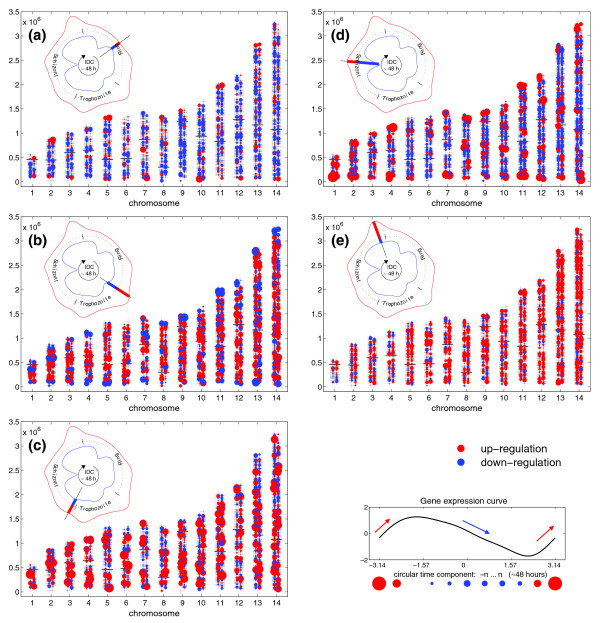
Snapshots of expression change rates during the IDC. Shown are five key frames of an animation of expression change rates during the IDC where red dots indicate up-regulation and blue dots down-regulation as determined by the rate of expression change. **(a) **After 8 hours at the beginning of the cycle (ring stage) the central chromosomal regions show an overall down-regulation (blue) while few genes within the subtelomeres are strongly up-regulated (red). **(b) **After 20 hours (late ring to early trophozoite stage) this is followed by an overall up-regulation in the center regions together with a down-regulation at the chromosomal ends. **(c) **After 30 hours (late-trophozoite to early-schizont), there is a mixture of strong up-regulation and weak down-regulation over the whole genome without specific subtelomeric activities. **(d) **After 40 hours (mid-schizont stage) a second strong up-regulation at the chromosome ends can be observed, which is accompanied by weak down-regulation in the intrachromosomal sections. **(e) **After 44 hour there is, again, an overall up-regulation in central chromosomal regions. Note the alternation of gene regulation between subtelomeric regions and central chromosomal regions. Upper left corner of each frame: 'infection timer' where the red and blue curves represent the number of genes having the strongest up- (red) or down- (blue) regulation at a specific time (density curves over time of strongest positive or negative change of expression; see also Materials and methods).

We further computed the first derivative of the gene expression functions with respect to the time component and plotted these values in a similar manner in order to visualize chromosome loci of strongest up- and down-regulation. By focusing on the rate of change of expression, which is given by the slope of the gene expression curve, we can determine how fast a gene switches from low to high expression and vice versa. Figure 4 shows five frames extracted from an animation of transcriptional regulation on the chromosomes during the IDC. The rate of up- and down-regulation is marked by red and blue dots, respectively, where the diameter of the dot indicates the rate of change (for example, a large red dot means strong up-regulation). The temporal position within the malaria infection cycle is, again, illustrated by a 48 hour infection 'timer' in the upper left corner of each illustration (see above). We observe an alternation of gene regulation between subtelomeric regions and central chromosomal regions. At the beginning of the cycle, in the middle of the ring stage, the central chromosomal regions show an overall down-regulation (blue) while few genes of subtelomeric regions are strongly up-regulated (Figure 4a, red). This is followed by an overall up-regulation in the center regions together with a down-regulation at the chromosomal ends during the switch from ring to trophozoite stage (Figure 4b). At the switch from trophozoite to schizont formation, we observe a mixture of strongly up-regulating genes and weak down-regulation over the whole genome without specific subtelomeric activities (Figure 4c). At mid-schizont stage, again we observe a strong up-regulation at the chromosome ends and weak down-regulation in the central chromosome proportions (Figure 4d); however, the subtelomeric up-regulated genes are different from the ones up-regulated during ring-stage (see below). This is followed, again, by a global up-regulation including the central chromosomal regions (Figure 4e). The full animation is available in Additional data file 6.

**Figure 5:**
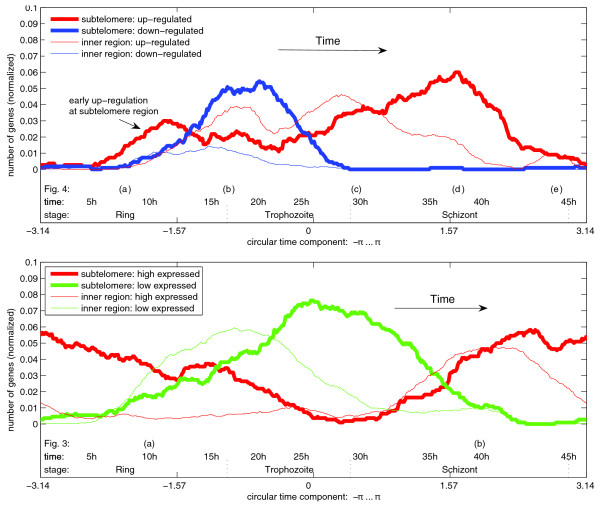
Histogram showing distinct regulatory events between subtelomere and inner chromosomal regions. For both subtelomere, up to 230,000 bp, and inner chromosomal regions, the number of up- and down-regulated genes (above) and the number of genes expressed at high or low levels (below) is plotted over time. Since inner chromosomal regions are larger than subtelomere regions, the gene counts were normalized (divided) by the total number of genes in subtelomere (1,100) and inner chromosomal regions (3,759). We used a threshold for both the rate-of-change graph and the expression level graph, and only consider the top 800 genes with strongest up-/down-regulation (above) or highest/lowest expression level (below). Note that highly regulated genes are not necessarily showing a high and low expression level, thus the genes counted above are not all identical with those counted below. Counting the genes confirms numerically that expression of genes of subtelomere regions is distinct from that of genes of central chromosomal regions (Figures 3 and 4). Top: up- and down-regulation (red and blue lines, respectively) of genes that are subtelomerically (bold line) or intrachromosomal (thin line) localized: while most subtelomere genes are up-regulated at the beginning (mid-ring) and end (mid-schizont) of the IDC, most intrachromosomal genes show an up-regulation in the trophozoite stage. Early up-regulation at subtelomeric regions is marked by an arrow. Bottom: high or low expression levels (red and green lines, respectively) of genes that are subtelomerically (bold line) or intrachromosomally (thin line) localized: most subtelomeric genes show a high expression level at late schizont/early ring. By contrast, most intrachromosomal genes are highly expressed only at early/mid-schizont stages.

**Figure 6:**
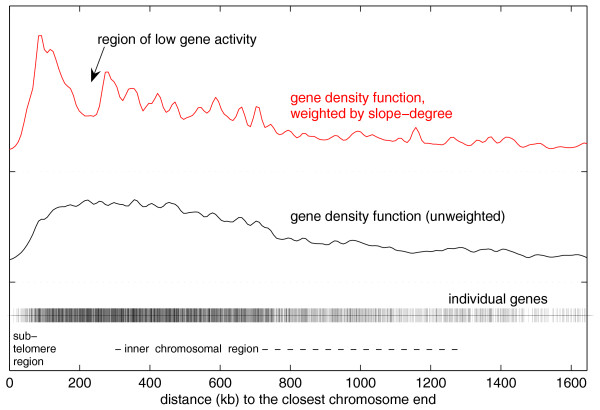
Gene activity with regard to distance from the telomeres. For comparison, all genes ('+') are plotted at the bottom of the figure on a linear scale representing the gene's distance to the closest chromosome end. The distribution of all genes with regard to the distance from the telomeres is shown by a density curve (black line), which is independent of the expression levels. After an increase of gene density at the telomeres, the curve shows an almost uniform distribution up to 500 kb that is followed by a smooth decrease caused by the different chromosome lengths. To take the rate of expression change into account, the contribution of each gene to the density curve is weighted by the fourth power of the maximal change rate of a gene (red curve, above). This weighted density curve shows a gene activity gap (at around 230 kb) that separates the subtelomeric regions that are strongly regulated early on in the IDC from the counter-regulated inner chromosomal regions.

To support these observations, histogram density curves were plotted in order to investigate if there is an accumulation of early activated genes at the subtelomeres (Figure 5). The histograms were calculated by counting the number of genes that are up- or down-regulated (Figure 5, upper panel, red and blue lines, respectively) or with high or low expression levels (Figure 5, lower panel, red and green lines, respectively) and that are located at intrachromosomal or subtelomeric regions (Figure 5, both panels, thin and bold lines, respectively). We used a threshold to limit the analysis to the 800 strongest regulating genes (genes with significantly changing expression levels). Inclusion of more genes will, in principle, lead to the same result, but due to inclusion of noisier data, the separation of the curves will be not as clear (data not shown). Thus, the density plots in Figure 5 validate our observation that genes that are activated early during the IDC are preferentially - though not exclusively - located in the subtelomeric areas of the malarial genome. To investigate this positioning bias further, we plotted rates of change with respect to chromosomal location. Figure 6 shows the gene density curve weighted by the maximal absolute change rate for each gene. To focus on the strongest up- und down-regulating genes, we used the fourth power of this rate of change. The results indicate a strong gene activity in subtelomeric regions and a comparably weak activity in central chromosomal regions. Between the subtelomeres and central regions there is a region of low gene activity (about 230,000 nucleotides from the telomeres), which suggests the presence of a 'boundary' between the two differentially regulated genome regions. This gene activity gap is also observed within the two other *P. falciparum *strains, 3D7 and Dd2 (data not shown). To summarize these observations, our model visualizes that early events of transcriptional activity take place at the subtelomeric regions before a global up-regulation of genes occurs.

**Figure 7:**
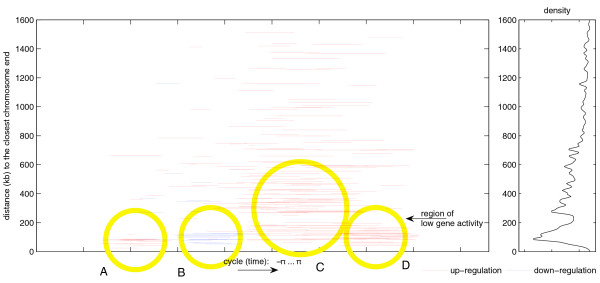
Change rates with respect to chromosomal location. By comparing change rates of the top 200 regulating genes and their respective chromosome positions, it becomes obvious that strongly regulating genes during early phases of the IDC are enriched in the periphery of the chromosomes (telomeres/sub-telomeres), whereas expression of central genes runs counter to telomeric regions. Genes are represented by red and/or blue lines, which refer to the time of up- (red) or down-regulation (blue). A threshold had to be applied for clarity of the figure and to focus on the strongest regulating genes. Here the threshold consists of an absolute slope degree larger than 3.5 (with respect to a cycle length of 2*π*; see also "Definitions" within Materials and Methods). The length of a line represents the duration wherein the expression rate strongly continuously increases or decreases. If the gene is only represented by a red line it means that the up-regulation was strong and above the threshold, but the down-regulation was too weak to be included in the graph. Note the four circled regions: A, with strong up-regulation at the subtelomeres (equivalent to class C1 in Figure 8); B, strongly down-regulated genes (belonging to classes C5 and C6 in Figure 8); C, genes that are localized throughout the genome and up-regulated around trophozoite stages (Figure 8, classes C2 and C3); D, second burst of up-regulation that mainly takes place in subtelomeric areas (Figure 8, class C5). This figure illustrates similar information as depicted in Figure 6, with the data now resolved over time. On the right-hand side the weighted density plot of Figure 6 is drawn as a reference. Again, the area of low gene activity is observed.

When displaying change rates and their respective chromosome positions (Figure 7), it becomes apparent that strongly regulating genes are enriched in the periphery of the chromosomes (subtelomeres), whereas expression of central genes runs counter to the subtelomeric regions. Figure 7 gives the distance of genes from the telomeres (y-axis) plotted against the time cycle (x-axis). After an initial phase of up-regulation at the subtelomeres, indicated by red horizontal lines up to approximately 100 kb from the telomeres, and followed by a down-regulation event (blue lines adjacent to the first 'red block'), a phase of overall strong gene activity is visible (cycle time 24 h to 36 h). However, one region seems to be excluded from this overall boost: regions of low gene activity are located at a distance of about 230,000 nucleotides from the telomeres (Figure 7, arrow).

### Highly regulated genes of subtelomeric and central chromosomal parts differ in their expression dynamics

**Figure 8:**
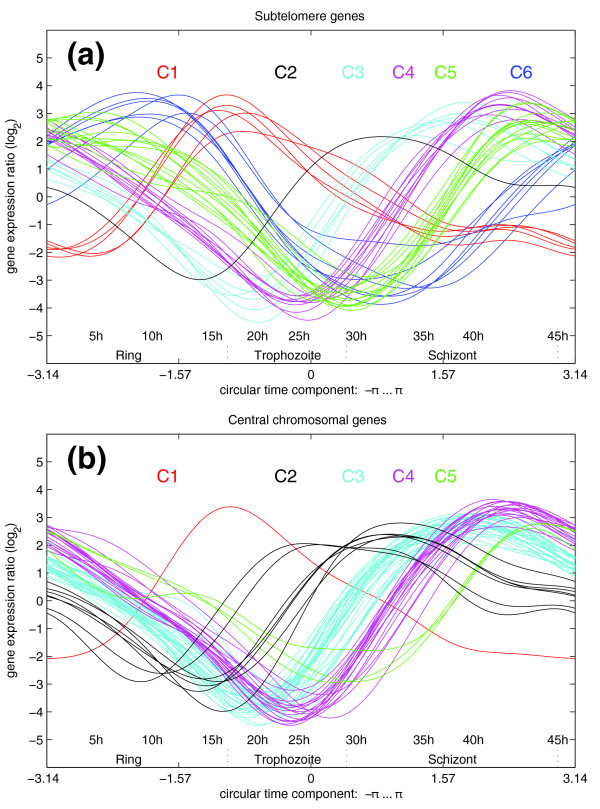
Comparison of expression patterns in subtelomeric and internal chromosomal regions. Displayed are expression curves of the top 100 genes with highest expression change rates. The genes were manually assigned to classes C1, C2, C3, C4, C5, and C6, which differ by their expression patterns. **(a) **Forty-two subtelomeric genes (position < 230 kb from the closest telomere). **(b) **Fifty-eight internal chromosomal region genes (>230 kb). Class C1 is over-represented in subtelomeric genes: only a single member of the C1 pattern (MAL7P1.58) is found amongst the top 100 intrachromosomal genes. By contrast, the C2 expression pattern is under-represented in subtelomeric genes, with PFI0160w being the only member of C2 in the subtelomeric proportion, whereas C2 is a rather common expression pattern in central chromosome parts. The class C6 expression pattern is solely observed in the subtelomeric areas. Similarly, the related pattern of class C5 is observed predominantly among subtelomeric genes. Class C3 and C4 patterns are present in both regions of the chromosomes, subtelomeric and central areas (see also the genome-wide up-regulation displayed, for example, in Figure 4c). Note the fast disappearance of the RNA in class C6 at the transition from ring to trophozoite stage, which might indicate unstable transcripts or active removal of message, whereas, for example, class C1 contains more stable mRNA as suggested by a low degree of down-regulation (approximately 25 h; see also the Discussion). Lists of genes are provided in Additional data files 2 and 3.

Figure 8 visualizes the subtelomeric and intrachromosomal genes with highest change rate of expression. For that analysis, we took into account the 100 strongest regulated genes of *P. falciparum *HB3 (considering both up- and down-regulation). Of these genes, 42 are located below a distance of 230,000 base pairs from a chromosomal end (Figure 8a, subtelomeric genes), whereas 58 are localized beyond that nucleotide threshold and, therefore, are regarded as 'central chromosomal' genes (Figure 8b). The genes have been manually classified into six gene-groups according to the time of strongest up-regulation (classes C1, C2, C3, C4, C5, and C6; indicated by different colors of the graphs). A list of genes in the respective profile groups is given in Additional data files 1-3.

The most interesting regulation characteristic is found in class C1, which shows the earliest upregulation during the IDC of the malaria parasite (approximately 10 h). Two genes of the C1 class contain a PHIST domain: MAL7P1.225 and PFI1785w. The functions of members of three subfamilies of PHIST proteins, PHISTa, PHISTb, and PHISTc, identified by Sargeant *et al*. [6] are currently not known. The authors speculate that the domains might contribute to a novel protein fold specific to *Plasmodium*. PHISTa proteins are entirely *P. falciparum *specific and the PHISTb subfamily has radiated extensively in *P. falciparum *in comparison to other *Plasmodium *species. Two PHISTb paralogs with a DNAJ domain (RESA and PF11_0509) are presumed to be part of an interaction network with skeleton-binding protein [6].

In other global transcriptional profiling experiments it has been shown that genes - now known to encode PHISTb and PHISTc proteins - are mainly active during early RBC stages [1,2,7], although a subset seems to be specifically up-regulated during gametogenesis [8,9], wherea *s *PHISTa (with the exception of PFD0090c and PFL2565w) genes have been shown to be transcriptionally silent in strain 3D7, which led Sargeant *et al*. to postulate that these genes also might be subject to mutually exclusive expression [6].

Pf332 (PF11_0506) and a PfMC-2TM pseudogene (PFB0960c) are also members of class C1. Most paralogs of the PfMC-2TM family have been found to be up-regulated in early gametocyte stages of *P. falciparum *development [6]. As gametocytogenesis is a break-out of the 'normal' cyclic intra-erythrocytic development, these data are not (or possibly cannot be) represented by our analysis. Interestingly, the only C1 member in a central chromosome location is a gene encoding a PfMC-2TM protein (MAL7P1.58), which suggests that it behaves cyclically in the malaria IDC. PFB0960c and PF10_0009, two other subtelomeric genes with a C1 class regulation dynamic, are annotated as pseudogenes but the strikingly strong regulation and the grouping within the subtelomeric regulation class C1 and a strong transcriptional signal hints at either a recent inactivation of the reading frames, rendering the genes as pseudogenes, or, alternatively, the general activation of the surrounding chromatin, which also would result in such a readout. Taken together, genes with a regulation dynamic of class C1, which shows strongest up-regulation at the earliest time during the IDC, are almost exclusively found at the subtelomeres.

In contrast to the C1 regulation dynamic, the C2 expression pattern is under-represented in subtelomeric genes. A single member of C2 is identified in the subtelomeric proportion of malaria chromosomes: a conserved protein of unknown function (PFI0160w). C2 is a rather common expression pattern in central chromosome parts and notably contains the putative cysteine proteases serine repeat antigen SERA-6 (PFB0335c) and SERA-5 (PFB0340c).

Class C3 and C4 patterns are present in both subtelomeric and central areas of the chromosomes and the up-regulation of genes belonging to these classes follows in a genome-wide boost after initial subtelomeric activity (C1). Subtelomeric C3 genes contain cytoadherence-linked asexual protein paralogs (MAL7P1.229 and PFI1730w), a PHIST domain protein (PF14_0732), and rhoptry-associated proteins RAP2 (PFE0080c) and RAP3 (PFE0075c). Genes encoding rhoptry associated proteins are also among the internal C3-type genes, such as those for RAP1 (PF14_0102), rhoptry neck protein 2 (PF14_0495), RhopH3 (PFI0265c), and high molecular weight rhoptry protein 2 (PFI1445w). The gene encoding merozoite surface protein 1 (MSP1, PFI1475w) is also found amongst the internal C3-type genes. Subtelomeric C4-type genes are mainly composed of adhesins such as erythrocyte binding antigens EBA181 (PFA0125c) and EBA175 (MAL7P1.176), reticulocyte-binding protein homologues (PFL2520w, PFD0110w, PFD1145c) or components involved in cytoskeleton formation or remodeling, like the membrane-skeletal protein IMC1-like proteins (PF10_0039 and PFC0185w) or coronin (PFL2460w). Internal C4-type genes for which an annotation is available seem to be heterogeneous in function.

A second round of regulation predominantly localized to the subtelomeres can be identified in C5 and C6 patterns, of which the class C6 expression pattern is solely observed in the subtelomeric areas.

Subtelomeric class C5, a class that shows up-regulation shortly before the second genome-wide up-regulation burst, contains proteins of unknown function, and, more interestingly, the etramp members 2 (PFB0120w), 14.1 (PF14_0016) and 11.1 (PF11_0039), two RESA paralogs that contain PHISTb and DNAJ domains (PFA0110w and PF11_0509), as well as an additional DNAJ domain containing protein (PF14_0013), and a gene for a putative efflux transporter (PF07_0004). etramp stands for early transcribed membrane protein and it should be noted in this context that we are analyzing the first derivative of gene expression curves and, thus, the up-regulation. Whereas up-regulation peaks in the schizont stage, the RNA is present early on during the IDC, giving rise to the appropriate nomenclature.

Class C6 expression levels are maximal at around 10 hours post-infection and show very strong down-regulation at the late ring/early trophozoite stage. The class contains membrane-associated histidine-rich protein (MAHRP-1, MAL13P1.413), proteins with unknown function (MAL13P1.61, PF13_0073), early transcribed membrane protein 10.1 (etramp 10.1, PF10_0019), a putative lysophospholipase (PF14_0017), ring exported protein (REX, PFI1735c), and an iRBC membrane protein (PFI1740c). Many proteins seem to be exported to the erythrocyte, as they carry a PEXEL motif, but an unbiased functional relationship of the class members is not obvious.

## Discussion

### Circular PCA can model intra-erythrocytic malaria parasite development

We analyzed the comprehensive malaria IDC transcriptome data from Bozdech *et al*. [1] and Llinas *et al*. [2] using circular PCA. In contrast to variable-wise smoothing algorithms, circular PCA is a multivariate analysis, meaning that it considers all variables (genes) at once. Such an integrated view interprets the dataset as a whole and takes dependencies between variables into account. Circular PCA is an unsupervised method. This means that the algorithm aims to identify the major information from the expression data alone, without using prior knowledge of the experimental set-up (here: time labels). In contrast to supervised regression models, where the time point would be explicitly supplied to the algorithm, an unsupervised data approximation model is exploratory, confirming that time is the most important factor in the datasets. The main variation of the data can be described by a single variable (circular component) that is related to time. The residual variation, which might be caused by other biological factors or technical artifacts, contributes to a much lower degree, which confirms that the experiments were well controlled. Since the time of the observed cycle is not exactly known, it would be difficult to supply a regression model with the right match between start and end time point, besides the problem of running the model in a circular manner. By contrast, using the unsupervised technique of circular PCA, the period of time is achieved as a result. The mapping of end time points to start time points is given by the data itself (Figure 2). Furthermore, the response time and developmental stage of individual organisms in any experiment differs from the exact physical time measurement. Hence, often we cannot absolutely trust the physical experimental time for the description of biological experiments. An unsupervised model, therefore, is superior in accommodating the unavoidable individual variability of biological samples.

In our analysis genes have been excluded that were constantly 'off' or 'on' or whose gene expression values did not exceed the noise level (see Materials and methods). It thus does not include analysis of genes that are exclusively expressed in the liver or mosquito stages that certainly are missed in such an analysis. For the remainder of the 3,639 genes of the *P. falciparum *HB3 dataset [1], we showed that a circular component exists (Figure 1), which we identified as the time component (Figure 2). The algorithm calculated the development cycle length to be about 47 hours, which fits the analyses of [1] and was able to confirm that time points 23 and 29 are missing (Figure 2). Our data model thus provides noise-reduced gene expression functions and even allows for interpolation of time points. The resulting gene expression functions are superior to using smoothing algorithms on gene expression curves, as we now can use the first derivative to calculate rates of expression change (up- and down-regulation).

### Early activation of transcription is located predominantly at the subtelomeric ends of chromosomes

**Figure 9:**
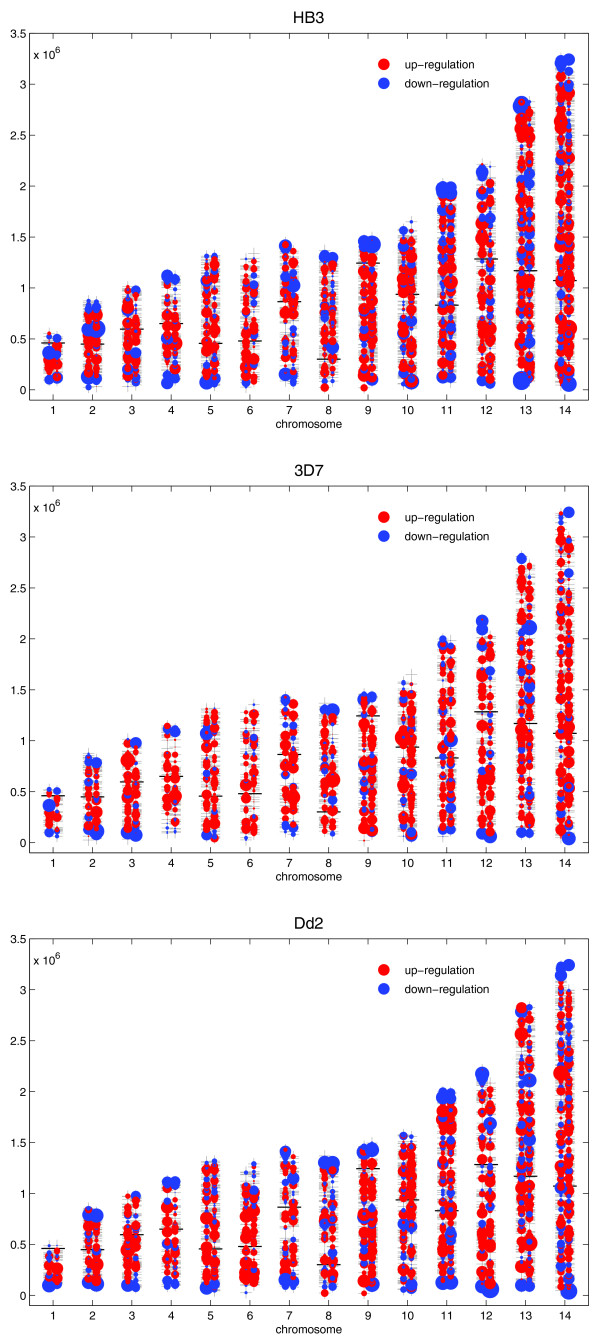
Snapshot of a comparison of the expression slopes of genes of *P. falciparum *strains HB3 (3,639 genes), 3D7 (2,419), and Dd2 (2,718) at approximately 22 h of the IDC. Genes were pre-filtered by quality criteria described in Material and methods. Note the similar characteristics of subtelomeric down-regulation and a burst of transcriptional activation throughout large parts of internal chromosome regions. Additional data file 4 lists the top 40 down-regulated genes of this comparative analysis for each of the analyzed strains.

Plotting either expression ratio levels (Figure 3 and Additional data file 5) or rates of change (Figure 4 and Additional data file 6) with respect to chromosomal position, we observed that early transcriptional events in the IDC take place at the subtelomeric regions and precede global transcriptional activities. Additional credence is given to this observation by analyzing expression data of two additional malaria strains, 3D7 and Dd2 [2], which behave similarly, although more genes had to be excluded from the analysis due to a higher noise level (Figure 9 and Additional data file 4).

Figure 9 illustrates a comparison of *P. falciparum *strains HB3, 3D7, and Dd2 (an exemplary snapshot taken at approximately 22 h of the developmental cycle), which has been included to indicate the similar processes on the subtelomeres of the three *P. falciparum *strains, which were analyzed with a similar time-resolution (HB3 [1]; 3D7 and Dd2 [2]). After pre-filtering (see Materials and methods) 3,639 genes (strain HB3), 2,419 genes (3D7), and 2,718 genes (Dd2) were subjected to circular PCA. In all three strains a strong down-regulation is observed in the subtelomeric areas of the chromosomes (blue dots), whereas genome-wide up-regulation is already initiating (red dots). Additional data file 4 lists the top 40 down-regulated genes of each of the investigated strains, HB3, 3D7, and Dd2. Due to an overlap between these datasets, the analysis resulted in a list including a total of 68 genes, of which 16 are shared between all three strains.

The 16 shared genes are those encoding the membrane associated histidine-rich protein MAHRP-1 (MAL13P1.413), ring exported protein REX (PFI1735c), EBA175 (MAL7P1.176), a putative interspersed repeat antigen (PFE0070w), tryptophan/threonine-rich antigen (PF08_0003), one PHIST domain protein (MAL8P1.4), the early transcribed membrane proteins ETRAMP10.1 (PF10_0019), 11.1 (PF11_0039), and 14.1 (PF14_0016), as well as ETRAMP2 (PFB0120w), proteins of unknown function that contain a predicted PEXEL trafficking motif (PFB0106c, MAL13P1.61, PFI1755c, and PF14_0760) as well as a conserved *P. falciparum *protein of unknown function (PFL0060w) and an - as of yet - hypothetical protein (PF11_0505). Further PHIST domain genes and other genes encoding members (for example, FIKK) of subtelomeric multigene families are identified in the residual data (Additional data file 4).

The overlap of early up-regulated genes between the investigated strains indicates that the observed early subtelomeric activity is a common phenomenon in malaria. Please also note that there is a second round of subtelomeric activity that precedes genome-wide transcriptional up-regulation during the schizont stage (for example, Figure 7d and Additional data file 6). Whereas the former group of genes belongs to class C1 (Figure 8), the latter group of genes belongs to class C5 (Figure 8). This lack of overlap of genes during the first and second round of subtelomeric upregulation leads us to hypothesize that the bias between terminal and central chromosome parts in *P. falciparum *chromosomes could be due to chromosomal architecture or position rather than promoter-driven gene-specific transcriptional activity.

In their analyses, Le Roch *et al*. [7] found a cluster of 95 subtelomeric genes expressed during early ring stage and late schizont stage that have been hypothesized to play important roles in establishing parasitemia within RBCs by remodeling the infected erythrocyte, which is confirmed by our algorithm. Circular PCA classifies these genes in classes C5 and C6 (Figure 8). The overlap of our results with the data from [7], which was gained using a different analysis platform and was analyzed by data clustering, gives additional credence to our findings.

In order to determine if a gene activity bias exists between subtelomeric and internal chromosome regions, we plotted up- and down-regulation with respect to chromosomal position for the top 200 regulated genes (Figure 7). Between the strongly regulating genes of the subtelomeres and the central chromosome parts, a gap was revealed (indicated by the arrow in Figure 7) in which no strongly regulated genes are present, although gene density in this area is normal (Figure 6). This gap could also be identified in the other two transcriptionally profiled *P. falciparum *strains, 3D7 and Dd2 (data not shown).

In malaria, chromosomal positioning effects have been described and are implied in antigenic variation of *P. falciparum*. The telomeres of malaria nuclear chromosomes associate in four to seven clusters in the nuclear periphery and contain a repeat-rich region that has been shown to be in a non-nucleosomal complex, while the region centromeric to the repeat elements is assembled in nucleosomes (for example, [10]; reviewed in [11]). Within this nucleosomally organized region, multigene families are encoded. The *var *gene family is composed of about 60 members and has been thoroughly investigated. *var *genes encode a parasite protein, PfEMP1, that is deposited on the surface of the infected RBCs. In a clonal population of parasites only a single member of the *var *gene family is expressed. This mutually exclusive expression is involved in evasion of the host's immune response (for example, reviewed in [12]). Occasionally, expression switches to a different *var *family member and, therefore, infected RBCs display different antigens. Duraisingh *et al*. [13] recently showed that the epigenetic action of PfSir2, a histone deacetylase, is involved in chromosomal repositioning of subtelomeric genes from transcriptionally inactive to active compartments of the parasite's nucleus. This repositioning combined with the *var *promoter activity [14] leads to an exclusive expression of a single copy of *var*. Since only a few transcriptional activators have been identified in *Plasmodium *[15], this also might suggest that the parasite regulates general gene expression by additional epigenetic means (reviewed in [16]). To monitor global histone modifications, Cui *et al*. [17] used ChIP-Chip studies in which the researchers could show that modified histones are distributed over the complete genome and, therefore, are relevant in global transcriptional activity. In our computational analysis, early events of transcription are clearly visible on almost all subtelomeric regions, whereas most genes in the centromeric portions are found to be down-regulated on the transcript level at the same time point (10 h, Figure 4a). By contrast, the picture changes at 20 hours post-infection (Figure 4b). While most genes that were activated early in the IDC are now down-regulated, a transcriptional burst of a large proportion of the centromeric genes takes place (beyond a distance of 230,000 bp from the telomeres). This sequence of subtelomeric activity preceding a genome-wide up-regulation happens a second time during the IDC: in the mid-schizont stage (see, for example, Figure 7d). Our observations were supported by density plots (Figure 5). It will be interesting to investigate which factors are responsible for this peculiar activity bias. Since the telomeres are associated in clusters in the nuclear periphery and gene loci can enter transcriptionally active or silenced suborganellar areas [11,12], one could envision that chromosomal architecture is involved in this bias. While research has been focused only on the telomeres or chromosome-terminal subtelomeric regions harboring variant surface protein gene families, we propose that the positioning events may stretch far beyond these chromosomal areas up to 230,000 bp towards the chromosome centers, thus enclosing chromosomal areas that harbor most members of *Plasmodium*- or *Apicomplexa*-specific multigene-families, such as *var*, *rifin*, *stevor*, *PHIST*, and so on (Additional data file 7).

The general transcription burst starting a mid-trophozoite stage raises one problem for the parasite since not all proteins are necessary within the cell at this point in time. This suggests that, in addition to transcriptional regulation, *Plasmodium *has some superimposed mechanisms to regulate transcript or protein abundance. When comparing correlation of mRNA with protein abundance, Le Roch *et al*. [18] found that, on average, 55% of the analyzed mRNA/protein pairs were present in the same developmental stage. This indicated that expression is regulated on the RNA level for these genes. For the remainder of the investigated pairings, the researchers postulated that proteins are expressed with a delay: proteins could be measured in one stage, whereas the respective transcript could be detected in the preceding IDC phase. Therefore, Le Roch *et al*. [18] hypothesized that post-transcriptional mechanisms were governing protein levels. This post-transcriptional gene silencing has also been observed in the murine malaria species, *P. berghei *[19], which indicates that this form of gene silencing might be a general strategy of *Plasmodium*. Mair and others [20] were able to show that an RNA helicase is involved in translational repression in malaria: a process in which mRNA is stored in ribonucleoprotein complexes for translation at a later time and that had been known previously only in multicellular eukaryotes. Thus, translational repression provides one possible explanation that *Plasmodium *is able to synthesize proteins 'in time', whereas the transcripts are formed during a transcriptional burst in which a huge portion of the *P. falciparum *transcriptome is up-regulated together.

Since our model is strictly based on RNA-level data, it currently cannot shed light on translational processes. Our expression model suggests, however, that there are additional superimposed mechanisms involved in the regulation of RNA abundance: we are able to identify classes of transcripts that disappear at lower or higher rates as indicated by the degree of down-regulation of transcripts. For instance, flat downward slopes for class 1 in Figure 8a indicate slowly fading RNA levels. This is also observed in certain functional groups of genes like eukaryotic-type ribosomal proteins (data not shown). By contrast, other mRNAs disappear at faster rates (steep slopes), suggesting active removal or unstable transcripts (for example, class 6, Figure 8a). This observation is reminiscent of a recent analysis by Shock *et al*. [21], who monitored mRNA decay rates during the IDC on a whole-genome basis. The researchers identified a change of RNA decay rates throughout the IDC, indicating a prominent role of mRNA stability in post-transcriptional regulation. As our finding of different change rates does not yet exclude the influence of differential transcriptional activation, it will be interesting to investigate the contribution of each, active transcription and mRNA stability, by integrating the datasets of [1] and [21].

Interestingly, in our analyses we identified regions on the chromosomes that show less gene activity when compared to the respective chromosomal neighborhood and that seem to be predominantly located about 230,000 bp centromeric of the chromosome ends (Figures 6 and 7). Since the gene density in these areas is as high as in the respective neighboring areas of the chromosomes, it is stunning that strongly regulated genes are under-represented in this area. This could be due to a marked accumulation of genes that are involved in different life cycle stages. However, conclusive evidence for this improbable event is lacking: most genes embedded within these regions are annotated as 'hypothetical proteins', and no clear functional assignments are possible as of yet (data not shown). Again, chromosomal architecture or epigenetic factors might be involved. Future analyses, such as time-resolved ChIP-Chip data and fluorescence microscopy, might assist in resolving this peculiar behavior.

## Conclusion

**Figure 10:**
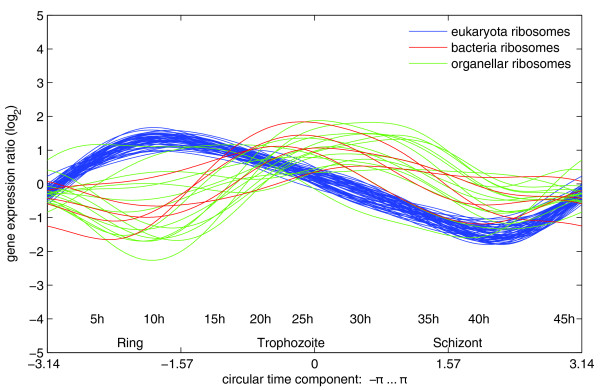
Regulation dynamics of genes encoding ribosomal proteins. Circular PCA-generated gene expression functions for *P. falciparum *HB3 genes encoding ribosomal proteins (*sensu Eukaryota*) follow a very similar regulation dynamic (blue curves), whereas genes belonging to organellar ribosomes are not as strictly controlled (red and green curves). Similar analyses might reveal candidates in which transcription initiation could be due to the action of transcriptional activators and might help understand control of gene expression in *P. falciparum *in the future.

We show that circular PCA, a special form of NLPCA, is capable of extracting a circular component from highly time-resolved malaria expression datasets. The resulting gene expression models allowed efficient noise reduction and time-point interpolation. Genome-wide analysis of derivatives of the resulting expression functions indicate that early up-regulation events originate at the subtelomeric regions of most of the 14 nuclear malaria chromosomes. Upon early activation of genes located at the subtelomeres, transcription activity is boosted genome-wide. This distinct behavior of the subtelomeres was identified within microarray data of three *Plasmodium *strains, HB3, 3D7, and Dd2, suggesting a general importance of malaria subtelomeres during the IDC. Our computational model fits hypotheses generated by the analysis of other research groups [1,18], which leads us to propose that circular PCA will assist in understanding regulatory processes of the malaria parasite development or any other model organism with highly dimensional data. As for malaria, lists of gene candidates obtained via circular PCA might be used to search for promoter motifs in upstream regions of genes with similar gene expression functions, the feasibility of which has been demonstrated previously [22,23]. One candidate target group for such an analysis could be genes encoding proteinaceous subunits of cytoplasmic ribosomes (*sensu Eukaryota*). Figure 10 illustrates the transcriptional regulation of genes encoding these ribosomal proteins by displaying the first derivative of circular PCA-generated gene expression functions for *P. falciparum *HB3. These genes follow a very similar regulation dynamic (blue curves), whereas genes belonging to organellar ribosomes are not as strictly controlled (red and green curves). Similar analyses might reveal candidates in which transcription initiation could be due to the action of transcriptional activators and might help increase understanding of control of gene expression in *P. falciparum *in the future.

Additionally, integration of expression data with multi-time point ChIP-Chip analyses or with mRNA decay experiments will enable us to make assumptions on the function and timing of the histone code or the contribution of RNA synthesis to RNA levels within the parasites, respectively. Further, by applying simple regulatory models on derivatives of the gene expression functions (monitoring rate-of-change rather than expression levels), identification of factors involved in transcriptional activation and silencing and construction of regulatory networks might prove possible in the future.

## Materials and methods

### Circular principal component analysis

Classic PCA [24] is a standard technique for dimensionality reduction. In molecular biology, PCA is widely applied to gene expression data for reducing the high dimensionality given by the large number of observed genes in order to visualize and interpret the data [25,26] or to use the reduced data of PCA for further analysis. While standard PCA is a linear technique that reduces the dataset by identifying a linear subspace that describes the data best in the mean square error sense, NLPCA, as a non-linear extension of PCA, is able to project the data onto a subspace that is curved (non-linear). The new variables that span the subspace are termed components, each of which is a combination of all original genes. The gene expression data of the intra-erythrocytic developmental cycle are supposed to follow a trajectory over time, meaning that the variation in the data is caused by only one factor, that is, time. Thus, the data can be well described by only one (non-linear) component. In principle, we have to find a single curve in the high-dimensional gene data space that approximates the data structure best, as shown in Figure 5. Since the IDC is a circular process, the data are supposed to form a circular structure and can be best described by a curve that is closed. For that we use circular PCA, a special type of non-linear PCA based on an artificial neural network algorithm (see [3,27] for more details).

The circular PCA algorithm is included in the non-linear PCA software package for MATLAB^® ^available at [28].

### Data acquisition and preprocessing

This analysis is based on microarray data of the *P. falciparum *HB3 strain from the experimental study of Bozdech *et al*. [1] accessible at the DeRisi Lab [29] and the *Plasmodium *genome [30] available via PlasmoDB [31]. Oligonucleotide positions were mapped to the genome by BLAST. Bozdech *et al*. monitored the IDC over 48 hours with a sampling time of 1 hour, thereby providing a series of time points: 1, 2,..., 48 [1]. Since two observations at 23 and 29 hours are missing, the total number of expression profiles (samples) is 46. The samples of individual time points (Cy5) were hybridized against a reference pool (Cy3), and the log2(Cy5/Cy3) ratios analyzed. Each gene is represented by one or more oligonucleotides on the microarray. Since oligonucleotides of the same gene show a similar expression [1], we combined them by using the median.

A controversy exists about the optimal data acquisition and normalization technique. While some studies consider the cumulative gene expression per cell [32], others normalize the data to constant total intensity such as the data of [1] used in our analysis. Periods of higher gene activity ('higher expression peak density') contribute a larger amount of RNA to the sample that is used for array studies. If normalization for constant total RNA (unit sum) is performed, the amount of the actual expression level of the investigated genes will be underestimated. Such artifacts are even more complicated in *P. falciparum *analyses as the amount of haploid genomes that contribute to expression grows exponentially within infected RBCs. In sum, both effects could lead to an artifactual cycling of expression log-ratios. It might seem tempting to use RNA abundance values as described in Martin *et al*. [32] in order to correct for this problematic situation; however, in how far a correction with values from one experimental set up [32] is beneficial for the analysis of literature data for another experimental set up (in this study from [1]) needs further discussion. We therefore introduced a quality criterion in order to reject 'noisy' genes (Additional data file 8). Since we expect expression curves to be smooth - that is, that the difference of expression values between two successive time points are small in relation to total variation over the whole time course - we reject expression values with drastic changes in neighboring time points. Even though there might be regulatory dynamics below the resolution of one hour, this cannot be considered in this study, since it would show a fluctuation that cannot be distinguished from noise. Also, genes that show no up- or down-regulation during the IDC will not be considered, as these result in a constant ratio around zero, which is sometimes corrupted by a large amount of noise in the data. Thus, we compared the distance of each expression value for each gene to its previous and next values, relative to the variance over all values. Expression values of distances three times larger than overall variance are removed and genes with more than 33% missing values are rejected.

The resulting dataset consists of log_2 _ratios of hybridizations of 3,639 genes at 46 time points. Identifying the optimal curve (circular component) in the very high-dimensional data space given by 3,639 individual genes is difficult or even intractable with 46 data points. Therefore, the 3,639 variables were linearly reduced to 12 principal components, each of which is a linear combination of all genes. The optimal number of principal components was determined by validating each number up to 30 by their missing data estimation performance as described in Additional data file 8. Applied to the reduced dataset, circular PCA provides us with a model of the IDC, which outputs the corresponding gene expression ratios at any chosen time point (also including interpolated time points). More detailed information is made available in Additional data file 8.

All figures were drawn using MATLAB^®^. The animations in Additional data files 5 and 6 were generated using the pdfanim latex package of Jochen Skupin [33].

### Definitions

#### Change rate (equivalent to 'slope')

Using the gene expression data, we generated a mathematical model of the intra-erythrocytic cycle, meaning continuous noise-reduced gene curves as functions over time, x = f(t), representing the dependencies of expression values x from component values (time) t for all genes. We further determined the slope of a gene expression curve at a time of interest by using the first derivative of this function with respect to time. Thus, the slope is the change rate of the gene expression curve.

What does a change rate or derivative of, for example, 3.5 mean? Due to the circular manner of the function, the cycle is given by values ranging from -π to π. With a cycle length of 2π for an assumed real duration of 47 hours, a change rate of 3.5 means an expression increase (log-ratio increase) of 3.5 × (2π/47) = 0.47 per hour.

#### Gene activity

Gene activity refers to the amount of actively expressed genes at a certain time interval. High gene activity therefore means that many genes are contributing to the total RNA of a cell.

#### 'Infection timers'

Infection timers in Figures 3 and 4 as well as in the animations in Additional data files 5 and 6 display the amount of genes that are expressed (high or low) or regulated (up or down). Red and green color in a graph or animation refers to high and low expression levels, respectively, whereas red and blue colors represent up- and down-regulation, respectively. The green inner curve of the infection timer in Figure 3, for example, indicates the amount of genes with low levels of expression, whereas the red, outer curve indicates the amount of highly expressed genes. The IDC starts at the top of the timer and proceeds through the 48 hours of intra-erythrocytic development of the malarial parasite. A red peak would indicate a period of high gene activity: many genes contribute to the total RNA of the parasite.

## Abbreviations

IDC, intra-erythrocytic developmental cycle; NLPCA, non-linear principal component analysis; PCA, principal component analysis; RBC, red blood cell.

## Authors' contributions

MS and MJF wrote the manuscript. MS performed data filtering and circular PCA. MJF performed data extraction, formatting, and mapping.

## Additional data files

The following Additional data are available with the online version of this paper. Additional data file 1 is a table listing all *P. falciparum *genes included in the circular PCA analysis. Additional data file 2 is a table listing all subtelomeric genes among the 100 top-regulated genes of *P. falciparum *HB3. Additional data file 3 is a table listing all internal genes among the 100 top-regulated genes of *P. falciparum *HB3. Additional data file 4 is a table listing 40 down-regulated genes with the steepest expression slopes resulting from a comparative analysis of *P. falciparum *strains HB3, 3D7, and Dd2 at approximately 22 h. Additional data file 5 is an animation of the IDC based on gene activities of strain *P. falciparum *HB3. Additional data file 6 is an animation of the IDC based on change of expression rates of strain *P. falciparum *HB3. Additional data file 7 is a figure illustrating the distances of members of *Plasmodium *multigene families from the telomeres. Additional data file 8 is a document that outlines additional methods and contains detailed information on the data pre-filtering process.

## Supplementary Material

Additional data file 1 *P. falciparum *genes included in the circular PCA analysis. Click here for file

Additional data file 2 Subtelomeric genes among the 100 top-regulated genes of *P. falciparum *HB3. Click here for file

Additional data file 3 Internal genes among the 100 top-regulated genes of *P. falciparum *HB3. Click here for file

Additional data file 4 Forty down-regulated genes with the steepest expression slopes resulting from a comparative analysis of *P. falciparum *strains HB3, 3D7, and Dd2 at approximately 22 h. Click here for file

Additional data file 5 Animation of the IDC based on gene activities of strain *P. falciparum *HB3. Click here for file

Additional data file 6 Animation of the IDC based on change of expression rates of strain *P. falciparum *HB3. Click here for file

Additional data file 7 Distances of members of *Plasmodium *multigene families from the telomeres. Click here for file

Additional data file 8 Additional methods and detailed information on the data pre-filtering process. Click here for file
