# Supplementary material for: A computational model of gene expression reveals early transcriptional events at the subtelomeric regions of the malaria parasite, Plasmodium falciparum
Source: Genome Biol. 2008 May 27;9(5):R88. doi: 10.1186/gb-2008-9-5-r88 (PMC2441474; doi:10.1186/gb-2008-9-5-r88)
Supplement: Additional data file 2 — Subtelomeric genes among the 100 top-regulated genes of P. falciparum HB3. [file gb-2008-9-5-r88-S2.pdf]

Matthias Scholz and Martin J. Fraunholz, *Genome Biology* (2008).
Additional data file 2

List of subtelomeric genes among 100 top regulated genes (see figure 8)

List of 42 subtelomeric genes among the 100 top-regulated genes of *P. falciparum* HB3. The subtelomeric genes were separated into classes C1, C2, C3, C4, C5, and C6 as shown in figure 8. Identifiers in parentheses relate to oligonucleotides that did not map to annotated genes. 'Distance', 'Start', and 'dir' indicate the distance from the closest telomere in nucleotides, the first nucleotide of the gene, and the strand, respectively.

group	gene	annotation	distance	chr	start	dir
C1	MAL7P1.225	PHISTa like protein, conserved in P. falciparum	80831	7	80831	-1
C1	PF10_0009	pseudogene-stevor, putative	57969	10	57969	1
C1	PF11_0507	Antigen 332	86137	11	1949113	1
C1	PFB0960c	Pfmc 2TM family protein, pseudogen	83718	2	863384	-1
C1	PFI1785w	PHIST domain protein	79335	9	1462388	1
C2	PFI0160w	conserved protein, unknown function	140976	9	140976	1
C3	MAL7P1.229	Cytoadherence linked asexual protein (CLAG)	110394	7	110394	-1
C3	PF14_0732	PHIST domain protein	152796	14	3138210	1
C3	PFE0075c	rhoptry-associated protein 3(RAP3)	81134	5	81134	-1
C3	PFE0080c	rhoptry-associated protein 2 (RAP2)	84041	5	84041	-1
C3	PFI1730w	Cytoadherence linked asexual protein (CLAG9)	127894	9	1413829	1
C3	(opff72505)	Upstream of PFF0200c: transcription factor with AP2 domains	179243	6	179243	1
C4	MAL7P1.176	erythrocyte binding antigen EBA175	88286	7	1413433	1
C4	PF08_0008	Conserved protein	181206	8	1238357	-1
C4	PF10_0039	Membrane-skeletal protein IMC1-like protein	164945	10	164945	1
C4	PF14_0044	Conserved Plasmodium protein, unknown function	161227	14	161227	-1
C4	PFA0125c	erythrocyte binding antigen EBA181	110984	1	110984	-1
C4	PFC0185w	Membrane-skeletal protein IMC1-like protein	201218	3	201218	1
C4	PFD0110w	reticulocyte-binding protein 1 homologue	144098	4	144098	1
C4	PFD1145c	reticulocyte-binding protein 5 homologue	117310	4	1086802	-1
C4	PFL2460w	Coronin (cytoskeletal organization)	179405	12	2092072	1
C4	PFL2520w	reticulocyte-binding protein 3 homologue	135440	12	2136037	1
C5	PF07_0004	Protein with multidrug efflux transporter domain	135268	7	135268	-1
C5	PF08_0003	Tryptophan/threonine-rich antigen	122358	8	1297205	-1
C5	PF11_0038	hypothetical protein	121435	11	121435	-1
C5	PF11_0039	early transcribed membrane protein etramp 11.1	126406	11	126406	1
C5	PF11_0509	ring-infected erythrocyte surface antigen, putative (RESA)	61933	11	1973317	1
C5	PF14_0013	DNAJ protein, putative	41290	14	41290	-1
C5	PF14_0016	Etramp 14.1	53412	14	53412	-1
C5	PF14_0045	conserved Plasmodium protein, unknown function	163990	14	163990	-1
C5	PFA0110w	ring-infected erythrocyte surface antigen precursor (RESA/PHIST/DNAJ domains)	99052	1	99052	1
C5	PFB0106c	Protein with unknown function and PEXEL domain	112547	2	112547	-1
C5	PFB0120w	early transcribed membrane protein etramp2	127994	2	127994	1
C5	PFD1170c	PHIST domain protein	87138	4	1116974	-1
C5	PFL0060w	conserved P. falciparum protein, unknown function	89934	12	89934	1
C6	MAL13P1.413	membrane associated histidine-rich protein (MAHRP-1)	111299	13	2784306	1
C6	MAL13P1.61	Protein with unknown function and PEXEL domain	99651	13	99651	1
C6	PF10_0019	early transcribed membrane protein etram 10.1	81417	10	81417	-1
C6	PF13_0073	conserved P. falciparum protein, unknown function	82821	13	82821	1
C6	PF14_0017	Lysophospholipase, putative	57173	14	57173	-1
C6	PFI1735c	Ring exported protein (REX)	121251	9	1420472	-1
C6	PFI1740c	iRBC membrane protein	114260	9	1427463	-1
