# Supplementary material for: A computational model of gene expression reveals early transcriptional events at the subtelomeric regions of the malaria parasite, Plasmodium falciparum
Source: Genome Biol. 2008 May 27;9(5):R88. doi: 10.1186/gb-2008-9-5-r88 (PMC2441474; doi:10.1186/gb-2008-9-5-r88)
Supplement: Additional data file 3 — Internal genes among the 100 top-regulated genes of P. falciparum HB3. [file gb-2008-9-5-r88-S3.pdf]

Matthias Scholz and Martin J. Fraunholz, *Genome Biology* (2008).
Additional data file 3

List of internal genes among 100 top regulated genes (see figure 8)

List of 58 internal genes among the 100 top-regulated genes of *P. falciparum* HB3. The genes were separated into classes C1, C2, C3, C4, and C5 as shown in figure 8. Identifiers in parentheses relate to oligonucleotides that did not match to annotated genes. 'Distance' gives the number of nucleotides from the closest telomere. 'Start' and 'dir' indicate the first nucleotide of the gene and the strand, respectively.

group	gene	annotation	distance	chr	start	dir
C1	MAL7P1.58	Pfmc-2TM Maurer's cleft two transmembrane protein	661033	7	661033	1
C2	PF11_0238	conserved Plasmodium protein, unknown function	865302	11	865302	-1
C2	PF13_0338	Pf92—cysteine-rich surface protein	330714	13	2564891	1
C2	PF14_0148	uracil-DNA glycosylase, putative	606378	14	606378	-1
C2	PFB0335c	cysteine protease, putative—serine repeat antigen 6 (SERA-6)	298897	2	298897	-1
C2	PFB0340c	cysteine protease, putative—serine repeat antigen 5 (SERA-5)	303593	2	303593	-1
C2	PFC0435w	conserved Plasmodium protein, unknown function	444174	3	444174	1
C2	PFL1725w	ATP synthase beta chain, mitochondrial precursor, putative	784406	12	1487071	1
C3	MAL13P1.130	conserved Plasmodium protein, unknown function	982812	13	982812	1
C3	MAL13P1.308	hypothetical protein, conserved	425742	13	2469863	1
C3	MAL7P1.119	hypothetical protein, conserved	499978	7	1001741	1
C3	MAL7P1.208	rifin-like protein	394422	7	394422	1
C3	MAL8P1.73	hypothetical protein, conserved	612640	8	806923	-1
C3	PF10_0119	hypothetical protein	470978	10	470978	1
C3	PF11_0194	hypothetical protein	708424	11	708424	-1
C3	PF11_0277	conserved Plasmodium protein, unknown function	997547	11	1037703	-1
C3	PF11_0373	conserved Plasmodium protein, unknown function	620873	11	1414377	-1
C3	PF13_0173	hypothetical protein, conserved	1332497	13	1332497	1
C3	PF13_0225	hypothetical protein, conserved	1255957	13	1639648	1
C3	PF14_0102	rhoptry-associated protein 1, RAP1	420441	14	420441	1
C3	PF14_0119	p1/s1 nuclease, putative	482436	14	482436	1
C3	PF14_0224	PP1-like protein serine/threonine phosphatase	936312	14	936312	1
C3	PF14_0325	ion channel, putative	1374579	14	1374579	-1
C3	PF14_0353	hypothetical protein	1512893	14	1512893	-1
C3	PF14_0495	rhoptry neck protein 2, putative	1157759	14	2133247	1
C3	PF14_0607	hypothetical protein	707258	14	2583748	-1
C3	PFB0670c	conserved Plasmodium protein, unknown function	347273	2	599829	-1
C3	PFB0680w	conserved Plasmodium protein, unknown function	337015	2	610087	1
C3	PFC0560c	conserved Plasmodium protein, unknown function	517582	3	542505	-1
C3	PFD0230c	protease, putative	266919	4	266919	-1
C3	PFF0645c	integral membrane protein	540072	6	540072	-1
C3	PFF0870w	conserved Plasmodium protein, unknown function	659638	6	758606	1
C3	PFF1365c	HECT-domain (ubiquitin-transferase), putative	295436	6	1122808	-1
C3	PFI0265c	RhopH3	270738	9	270738	-1
C3	PFI1445w	High molecular weight rhoptry protein-2	366530	9	1175193	1
C3	PFI1475w	merozoite surface protein 1, precursor	339921	9	1201802	1
C3	(ks1704_1)		338377	11	338377	-1
C4	MAL13P1.260	hypothetical protein, conserved	839658	13	2055947	1
C4	PF07_0104	kinesin-like protein, putative	393876	7	1107843	1
C4	PF08_0108	pepsinogen, putative	417466	8	417466	-1
C4	PF10_0138	hypothetical protein	555159	10	555159	1
C4	PF10_0170	hypothetical protein	701745	10	701745	1
C4	PF10_0295	hypothetical protein	462642	10	1231803	-1
C4	PF10_0346	merozoite surface protein 6	285928	10	1408517	1
C4	PF13_0058	RNA-binding protein, putative	468434	13	468434	-1
C4	PF13_0211	calcium-dependent protein kinase, putative	1366908	13	1528697	-1
C4	PF14_0527	hypothetical protein	1032592	14	2258414	1
C4	PFA0440w	hypothetical protein, conserved	278821	1	364471	1
C4	PFB0665w	serine/threonine protein kinase, putative	352906	2	594196	1
C4	PFC0355c	hypothetical protein	362334	3	362334	-1
C4	PFC0830w	trophozoite stage antigen	274963	3	785124	1
C4	PFE1285w	membrane skeletal protein IMC1-related	272309	5	1071243	1
C4	PFL1945c	early transcribed membrane protein 12, ETRAMP12	586395	12	1685082	-1
C4	(opfa32034)		277917	1	365375	1
C4	(opfblob0018)		363402	3	363402	-1
C5	MAL8P1.88	hypothetical protein, conserved	701497	8	701497	1
C5	PF08_0072	hypothetical protein, conserved	707197	8	707197	1
C5	PF11_0166	hypothetical protein	593903	11	593903	-1
