# Supplementary material for: A computational model of gene expression reveals early transcriptional events at the subtelomeric regions of the malaria parasite, Plasmodium falciparum
Source: Genome Biol. 2008 May 27;9(5):R88. doi: 10.1186/gb-2008-9-5-r88 (PMC2441474; doi:10.1186/gb-2008-9-5-r88)
Supplement: Additional data file 5 — Animation of the IDC based on gene activities of strain P. falciparum HB3. [file gb-2008-9-5-r88-S5.pdf]

Animation of the intraerythrocytic development cycle based on gene activities of the *P. falciparum* genome

Description: The animation of the intraerythrocytic development cycle is based on gene activities of genes on the 14 nuclear chromosomes of *P. falciparum* HB3 (see also figure 3). The upper left corner displays an 'infection timer' which indicates high (red) and low (green) expression of all genes during a certain time point. The genes with strong (red dots) or weak (green dots) expression are indicated, while the diameter of the dot indicates the expression level (e.g., large green dot means very low expression). As a result we observed that subtelomeric regions show a behaviour which is clearly distinct from central chromosomal regions.

Click 'Play' on the next page to start the animation.

Matthias Scholz and Martin J. Fraunholz, *Genome Biology* (2008).

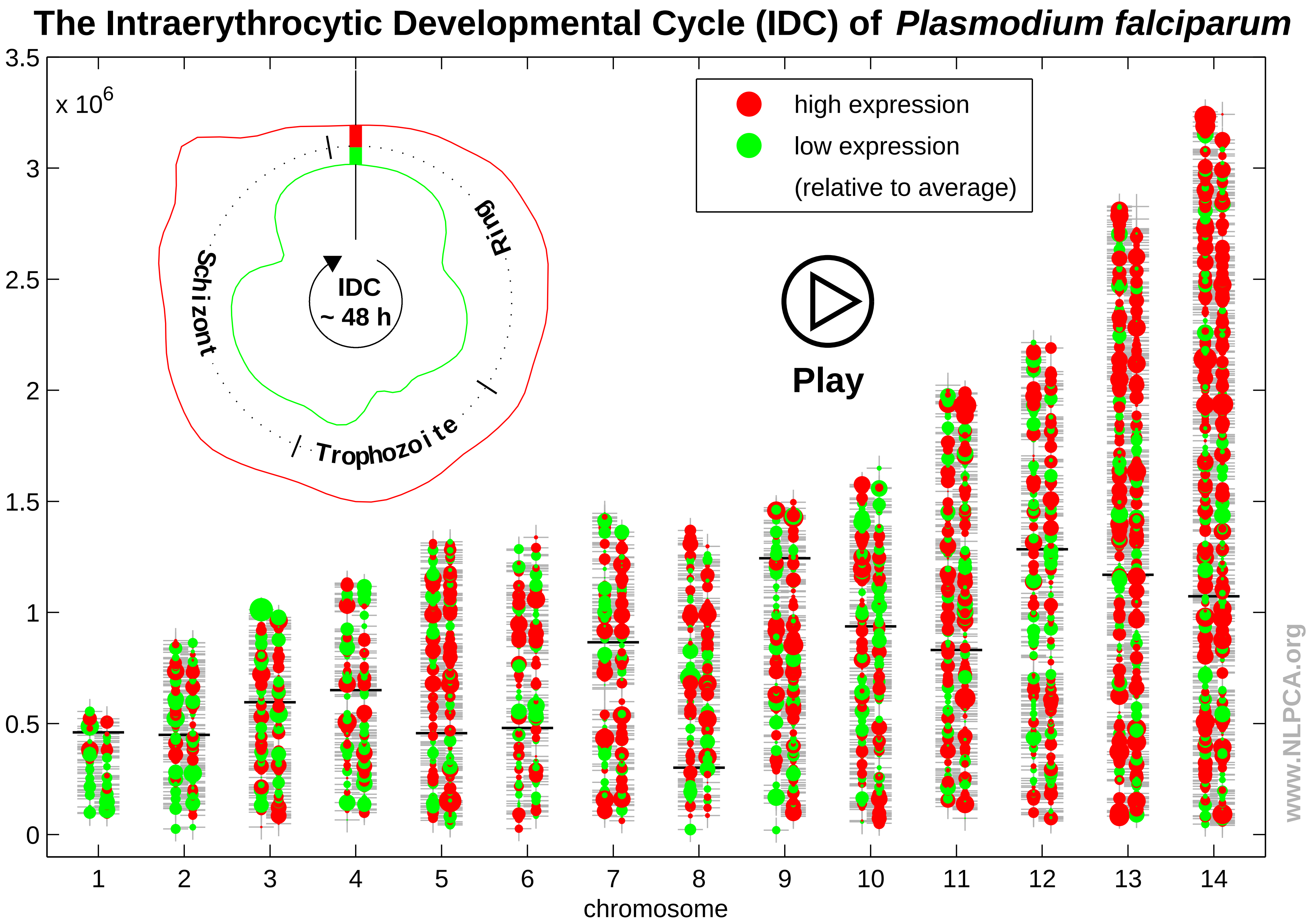

The Intraerythrocytic Developmental Cycle (IDC) of *Plasmodium falciparum*
x 10^6
3.5
3
2.5
2
1.5
1
0.5
0
high expression
low expression
(relative to average)
Ring
Schizont
Trophozoite
IDC
~ 48 h
Play
1
2
3
4
5
6
7
8
9
10
11
12
13
14
chromosome
www.NLPCA.org
