# Supplementary material for: A computational model of gene expression reveals early transcriptional events at the subtelomeric regions of the malaria parasite, Plasmodium falciparum
Source: Genome Biol. 2008 May 27;9(5):R88. doi: 10.1186/gb-2008-9-5-r88 (PMC2441474; doi:10.1186/gb-2008-9-5-r88)

Animation of the intraerythrocytic development cycle based on change of expression rates of the *P. falciparum* genome

Description: This animation of the intraerythrocytic development cycle is based on change of expression rates of genes on the 14 nuclear chromosomes of *P. falciparum* HB3 (see also figure 4). The upper left corner displays an 'infection timer' which indicates up- (red) and down regulation (blue) of all genes during a certain time point. The genes with strong up (red dots) or down-regulation (blue dots) are indicated. The diameter of the dot indicates the degree of regulation (e.g., large red dot means strong up-regulation).

Click 'Play' on the next page to start the animation.

Matthias Scholz and Martin J. Fraunholz, *Genome Biology* (2008).

The Intraerythrocytic Developmental Cycle (IDC) of *Plasmodium falciparum*

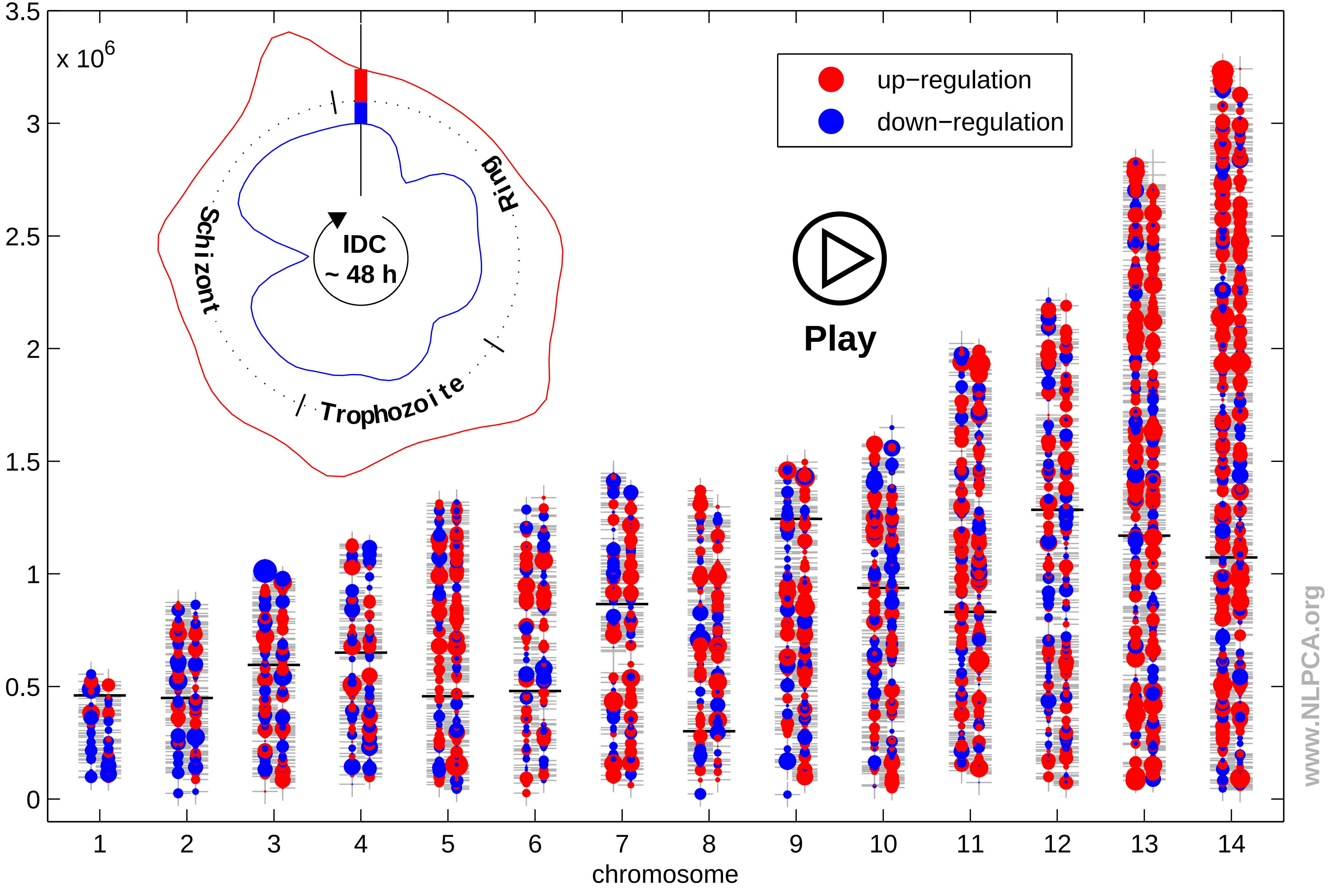

Supplement: Additional data file 6 — Animation of the IDC based on change of expression rates of strain P. falciparum HB3. [file gb-2008-9-5-r88-S6.pdf]
