# Supplementary material for: A computational model of gene expression reveals early transcriptional events at the subtelomeric regions of the malaria parasite, Plasmodium falciparum
Source: Genome Biol. 2008 May 27;9(5):R88. doi: 10.1186/gb-2008-9-5-r88 (PMC2441474; doi:10.1186/gb-2008-9-5-r88)
Supplement: Additional data file 7 — Distances of members of Plasmodium multigene families from the telomeres. [file gb-2008-9-5-r88-S7.pdf]

Matthias Scholz and Martin J. Fraunholz, *Genome Biology* (2008).
Additional data file 7

Distance from the telomeres of members from different multigene families

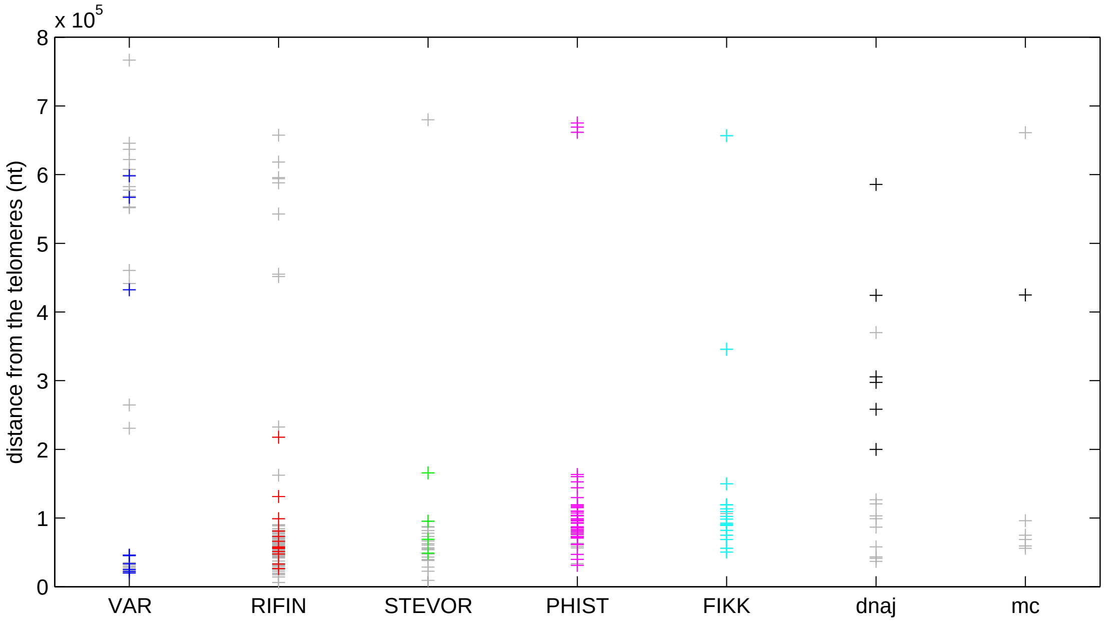

Illustrated is the distance of members of *Plasmodium* multigene families from the telomeres. Family members that showed significant regulation levels in the circular PCA analysis of the *P. falciparum* HB3 transcriptome are displayed in color. Plotted in gray are the positions of the gene family members that did not enter the circular PCA analysis due to a signal-to-noise threshold [see also Additional data file 8]. Most members of *var*, *rifin*, *stevor*, Pfmc-2TM (mc), DnaJ-domain, and PHIST-domain proteins are located within the first ∼200,000 subtelomeric nucleotides. Please note that this span approximately corresponds to our analysis of gene loci showing early upregulation (e.g., as shown in figures 5, 6, and 7). Distances are given in 10^5 nucleotides.
