# Supplementary material for: A computational model of gene expression reveals early transcriptional events at the subtelomeric regions of the malaria parasite, Plasmodium falciparum
Source: Genome Biol. 2008 May 27;9(5):R88. doi: 10.1186/gb-2008-9-5-r88 (PMC2441474; doi:10.1186/gb-2008-9-5-r88)
Supplement: Additional data file 8 — Additional methods and detailed information on the data pre-filtering process. [file gb-2008-9-5-r88-S8.pdf]

Matthias Scholz and Martin J. Fraunholz.
A computational model of gene expression reveals early transcriptional events at the subtelomeric regions of the malaria parasite, *Plasmodium falciparum*. *Genome Biology* (2008).

Additional data file 8: Supplementary methods

Expression quality criterion

The expression quality criterion is defined as follows: In order to detect outliers, we compare the distance from one gene expression value x_t to the next and previous value (local variation) with the overall variation of all observations of gene x. The overall variation β is given by the variance over all N observed expression values of a gene: $\beta = \frac{1}{N}\sum_{t=1}^{N}(x_t - \bar{x})^2$ with mean $\bar{x}$. The local variation α_t of observation x_t at time t includes the previous x_{t-1} and next x_{t+1} observations and is given by the squared distance $\alpha_t = \frac{1}{2}\sum_{\tau=[-1,+1]}(x_{t+\tau} - x_t)^2$. The local variation α_t is set into relation to the overall variance β by $c_t = \frac{\alpha}{\beta}$. If the criterion c_t exceed a threshold of 3.0, the corresponding observation x_t regards as an outlier and is rejected from the data set (set as missing). Thus expression values of distances three times larger than overall variance are rejected. Finally, genes with more than 33% missing values (original missing values as well as rejected outliers) are removed.

Estimating the optimal number of components in PCA pre-processing

The high-dimensional data set of 3,639 genes is linearly reduced to a much smaller component set by standard (linear) principal component analysis (PCA). The optimal reduced dimensionality (the number of principal components) was achieved by a validation based on missing data estimation performance at different numbers of components. As shown in Figure 1, the best (minimal) value was achieved at 12 principal components.

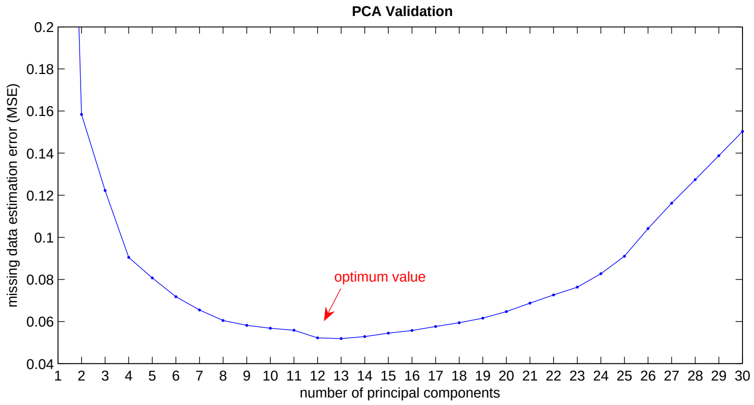

Figure 1: Validation by missing data estimation performance of PCA. Different numbers of principal components were used to estimate artificially removed values (two values per gene, 4%). Shown is the median of the mean square estimation error (MSE) over 100 runs. The optimal number of principal components is 12 (shown by an arrow).

To estimate missing data in a linear PCA manner, we need an PCA algorithm that handles missing data. For that we used the linear mode of the NLPCA neural network working in inverse mode [5]. Alternatively, *probabilistic PCA* (PPCA) [34] can be used to estimate missing values by (linear) PCA. A MATLAB® implementation of *probabilistic PCA* for missing values is available by Jakob Verbeek [35].
